# Spatial Pattern and Associated Factors of ANC Visits in Ethiopia: Spatial and Multilevel Modeling of Ethiopian Demographic Health Survey Data

**DOI:** 10.1155/2020/4676591

**Published:** 2020-08-19

**Authors:** Zemenu Tadesse Tessema, Temesgen Yihunie Akalu

**Affiliations:** Department of Epidemiology and Biostatistics, Institute of Public Health, College of Medicine and Health Sciences, University of Gondar, Gondar, Ethiopia

## Abstract

**Background:**

Although there is an increase in having antenatal care (ANC), still many women lack recommended ANC contacts in Ethiopia. Therefore, this study was aimed at determining spatial patterns and associated factors of not having ANC visits using the Ethiopian Demographic and Health Survey (EDHS) 2016 data.

**Methods:**

A two-stage stratified cluster sampling technique was employed based on EDHS data from January 18 to June 27, 2016. A total of 7,462 women were included in the study. ArcGIS version 10.7 software was used to visualize the spatial distribution. The Bernoulli model was applied using Kilduff SaTScan version 9.6 software to identify significant purely spatial clusters for not having ANC visits in Ethiopia. A multivariable multilevel logistic regression model was used to identify individual- and community-level determinants of not having antenatal care. Model comparison was checked using the likelihood test and goodness of fit was assessed by the deviance test.

**Results:**

The primary clusters' spatial window was located in Somalia, Oromia, Afar, Dire Dawa, and Harari regions with the log-likelihood ratio (LLR) of 133.02, at *p* < 0.001 level of significance. In this study, Islam religion (adjusted odds ratio (AOR) = 0.7 with 95% confidence interval (CI) (0.52,0.96)), mother education being primary (AOR = 0.59, 95% CI (0.49,0.71)), distance from health facility being a big problem (AOR = 0.76, CI (0.65,0.89)), second birth order (AOR = 1.35, CI (1.03, 1.76)), richer wealth index (AOR = 0.65, CI (0.51,0.82)), rural residence (AOR = 2.38, CI (1.54,3.66)), and high community media exposure (AOR = 0.68, CI (0.52,0.89)) were determinants of not having antenatal care in Ethiopia.

**Conclusion:**

The spatial distribution of ANC in Ethiopia is non-random. A higher proportion of not having ANC is found in northeast Amhara, west Benishangul Gumuz, Somali, Afar, north, and northeast SNNPR. On the other hand, a low proportion of not having ANC was found in Tigray, Addis Ababa, and Dire Dawa. In Ethiopia, not having antenatal care is affected by both individual- and community-level factors. Prompt attention by the Federal Ministry of Health is compulsory to improve ANC especially in rural residents, uneducated women, poor households, and regions like Oromia, Gambella, and Somalia.

## 1. Background

Antenatal care (ANC) is a vital opportunity to both the mother and infant through diagnosing and treating pregnancy-related complications and by providing direct interventions [1]. ANC coverage is an indicator to assess progress towards the Millennium Development Goals and an opening to reach fundamental interventions [2]. According to the World Health Organization (WHO), improving the quality of ANC by increasing the number of recommended contacts (a minimum of 4 times) and early booking (12 weeks) can reduce the risk of stillbirth and maternal complications [3]. According to WHO 2015 report, nearly 303,000 women and adolescent girls died from pregnancy and childbirth-related complications and about 2.6 million babies were stillbirths [4]. Unfortunately, 99% of maternal deaths and 98% of child deaths occurred in low- and middle-income countries. If pregnant women gained ANC, these maternal deaths could be prevented [5]. Of all deaths, 1.46 million (60%) occurred due to untreated maternal infection, poor fetal growth, and hypertension disorders of pregnancy [6].

According to EDHS 2016, the proportion of women, 15–49 years, who received ANC by skilled birth attendants was 62%. The trend analysis showed that the increase in ANC coverage was to 27%, 28%, 34%, and 62%, in 2000, 2005, 2011, and 2016, respectively. Among regions, ANC coverage was highest in Addis Ababa (97%) and lowest in the Somali region (44%) [7].

ANC utilization can be affected by sociodemographic and economic factors such as educational status of mothers [8–10], rural residency [9, 11], wealth index [10, 11], religion [12], regional variation [13], maternal factors and child factors like presence of the previous history of low birth weight (LBW) child [14], history of preterm birth [15], maternal death [6], planned pregnancy [8], history of stillbirth [8], birth order [16], early age [17], unwanted pregnancy [18], and intimate partner violence [19], and inequality in maternal service delivery and existing health system [20, 21]. Moreover, a systematic review and meta-analysis in Sub-Saharan Africa showed that socioeconomic factors (residence, age, maternal education, partner education, occupational status, marital status, not having health insurance, and religion), maternal factors (parity, having an unplanned pregnancy, previous pregnancy complications, awareness of danger signs, timing, and an adequate number of previous antenatal visits), exposure to mass media, attitude towards ANC utilization, autonomy, husband's support, distance to a health facility, and cost of services affect ANC utilization [22].

Different strategies were tried to improve the ANC coverage. For instance, WHO encourages 8 contacts for every pregnant woman and recommends the clinician to provide necessary information during contact. Besides, WHO recommends community members to directly participate in providing information on the importance of ANC to pregnant women at each contact [23].

Although there is a reduction in not having ANC, a spatial pattern for not having ANC visits was still unknown and many women lack recommended ANC contacts in Ethiopia. Therefore, this study was aimed at determining spatial patterns and individual- and community-level factors for not having ANC visits in Ethiopia.

## 2. Methods

### 2.1. Study Design, Period, and Setting

The population-based cross-sectional study design was conducted in Ethiopia from January 18 to June 27, 2016. Ethiopia is located in the Horn of Africa and has 9 regional states and two-city administrations.

### 2.2. Data Source

The analysis was based on the 2016 EDHS women data set. The approval letter was gained from the Measure DHS and data were downloaded from the Measure DHS website, http://www.measuredhs. The survey includes all nine regions and the two-city administration of Ethiopia. Participants were selected based on a stratified two-stage cluster sampling technique. The full details of methods and procedures used in data collection in the EDHS have been published elsewhere [7]. The survey collects information from a national representative sample of 15,683 women, age 15–49. Finally, 7,462 women were included in this study.

### 2.3. Study Variable

The outcome variable, ANC visit, was a binary variable, categorized as having ANC visits or not. Women who had at least one ANC visit five years preceding the survey by trained medical personnel were categorized as having ANC visits and coded as zero and women who had no ANC follow-up were classified as not having ANC follow-up and coded as 1. Explanatory variables were assessed at the individual and community levels to address the multilevel effect on not having ANC visits. Individual-level factors include the age of women, religion, women's educational status, husband education, sex of household head, mother occupation, husband occupation, wealth index, media exposure, birth order, distance from the health institution, parity, and marital status, whereas community-level factors include region, place of residence, community distance to a health facility, community poverty, community women's education, and community media exposure. Among community-level factors, place of residence and region were directly collected to explain the characteristics of clusters, whereas other community-level variables were generated by aggregating individual-level characteristics at the community (cluster) level and categorization of the aggregate variables was done as high or low based on 50% values calculated for each community (community-level education was aggregated as low if a proportion of women were educated below 50% and high if the proportion is ≥ 50%, community poverty was aggregated as low if the proportion of women from the wealth quartile in a given community is <50% and as high if the proportion is ≥ 50%, community media exposure was aggregated as high if the proportion of the community exposed to media is at least 50% and low if <50%, and community distance to the health facility was aggregated as a big problem if the proportion of women in the given community who perceived it as far is at least 50% and not a big problem if it is <50%.

### 2.4. Spatial Autocorrelation Analysis

The spatial autocorrelation (Global Moran's I) statistic measures whether an ANC visit was dispersed, clustered, or randomly distributed in the study area [1]. Moran's I is a spatial statistics measure used to measure spatial autocorrelation by taking the entire data set and producing a single output value which ranges from −1 to +1. Moran's I values close to −1 indicate disease dispersed, whereas I close to +1 indicates disease clustered and disease distributed randomly if I value is zero. A statistically significant Moran's I (*p* < 0.05) leads to rejection of the null hypothesis (ANC visit is randomly distributed) and indicates the presence of the spatial autocorrelation [24].

### 2.5. Incremental Autocorrelation

This measures spatial autocorrelation for a series of distances and optionally creates a line graph of those distances and their corresponding z-scores. Z-scores reflect the intensity of spatial clustering, and statistically significant peak z-scores indicate distances where spatial processes promoting clustering are most pronounced. These peak distances are often appropriate values to use for tools with a distance band or distance radius parameter. This tool can help you select an appropriate distance threshold or radius for tools that have these parameters, such as hot spot analysis [1].

### 2.6. Hot Spot Analysis (Getis-Ord Gi^*∗*^ Statistics)

Getis-Ord Gi^*∗*^ statistics were computed to measure how spatial autocorrelation varies over the study location by calculating GI^*∗*^ statistic for each area. Z-score was computed to determine the statistical significance of clustering, and the *p*-value computed for the significance. Statistical output with high GI^*∗*^ indicates “hot spot” whereas low GI^*∗*^ means a “cold spot.”

### 2.7. Spatial Interpolation

The spatial interpolation technique was used to predict ANC visits on the unsampled areas in the country based on sampled EAs. Ordinary Kriging spatial interpolation method was used for this study for predictions of not having ANC visits in unobserved areas of Ethiopia.

### 2.8. Spatial Scan Statistical Analysis

A Bernoulli-based model was used in which events at particular places were analyzed, whether having ANC visits or not as 1/0. The scan statistics developed by Kulldorff and SaTScan™ software version 9.6 were used to identify the presence of purely spatial ANC visit clusters. Scan statistics did scanning gradually across the space to identify the number of observed and expected observations inside the window at each location. The scanning window with the maximum likelihood was the most likely high performing clusters, and a *p*-value was assigned to this cluster [25].

### 2.9. Statistical Analysis

Data were analyzed using STATA version 14 software. Sampling weight was applied to keep population representation throughout enumeration areas. Descriptive statistics like tables and texts were used to explore study subjects. A multilevel multivariable logistic regression analysis was analyzed to account for the hierarchal structure [26–29]. First, bivariate multilevel logistic regression analysis was performed to find the crude odds ratio at a 95% confidence interval, and those variables statistically significant at 0.2 were used in the multilevel multivariable logistic regression analysis. Lastly, multilevel multivariable logistic regression analysis was performed to estimate the adjusted odds ratio and random variation between clusters. Statistically significant variables at *p*-value less than 0.05 were reported with their 95% confidence interval. Regarding missing data analysis deletion was used for respondents that missed the outcome variable and missing related to explanatory variables were analyzed as it is using the complete case analysis principle in STATA.

The null model (without incorporating any factors) was used to test the random effect (cluster variation on ANC) to estimate the Interclass Correlation Coefficient (ICC). The second model examined the effects of individual-level factors on women who have not had ANC visits. The 3^rd^ model contained only community-level factors. Furthermore, ICC was estimated and observed if there was a decline in the between-cluster variability upon adding community-level factors. Lastly, the 4^th^ model was examined by incorporating both the individual- and community level factors. Then, the best-fitted model was chosen using a likelihood ratio test. The goodness of fit was determined using the deviance test. Consequently, the model with the highest likelihood ratio test and the lowest deviance was chosen as the best-fitted model.

### 2.10. Parameter Estimation Methods

In the multilevel multivariable logistic regression model, fixed effect estimates measure the association between the odds of not having ANC visits of individual- and community-level factors with 95% confidence Interval. ICC quantifies the degree of heterogeneity of not having ANC visits between clusters [30]. The proportion of change in variance (PCV) measures the proportion of the total observed individual variation that is attributable to the between-cluster variations [31]. The median odds ratio (MOR) measures the value between high- and low-risk clusters (EAs) [32].

## 3. Results

### 3.1. Sociodemographic Characteristics of Respondents

A total of 7,462 women were included in the analysis with a response rate of 98%. The mean age of respondents was 29 years with SD of ±9.4 years. More than 60% of mothers were in the age group of 20–34 years. Almost all (>90%) of the women were married five years preceding the survey. Regarding the educational status of the mothers, 7,201 (66%) were unable to read and write. Moreover, 6,057 (56%) of the mothers had no work in the five years preceding the survey (Table 1).

### 3.2. Characteristics of the Cluster

In EDHS 2016, 645 clusters were selected. Of these, 643 clusters were eligible. The maximum number of households selected per cluster was 28. Among the total number of clusters, 69% were rural in residence and almost half (49%) of the clusters had a big problem in accessing any health institution. Among 643 clusters, half of them had low community women educational attainment. Nearly half (50.08%) of the community had media exposure (Table 2).

### 3.3. Spatial Distribution of Not Having ANC Utilization

**Figure 1:**
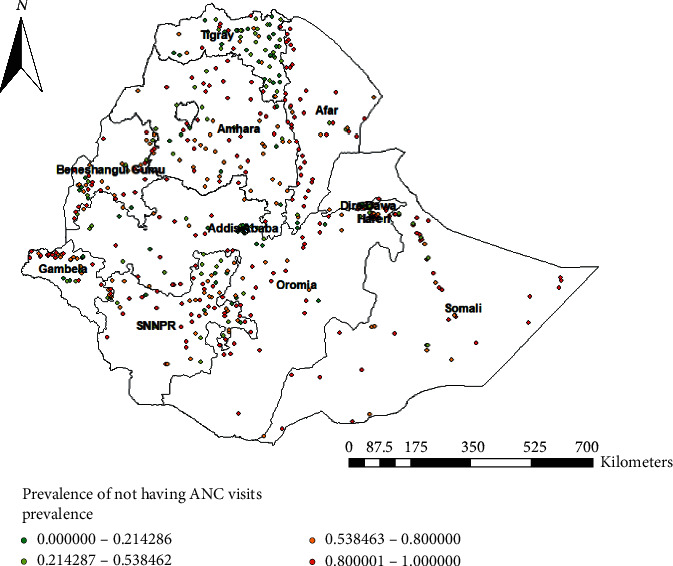
Spatial distribution of not having ANC visits across the country, EDHS 2016.

A total of 622 clusters were considered for the spatial analysis of not having ANC utilization. Each point on the map represents one enumeration area with the proportion of not having ANC utilization in each cluster. The red color indicates areas with a high proportion of not having ANC utilization whereas the green color indicates EAs with a low proportion of not having ANC utilization. A higher proportion of not having ANC utilization was found in northeast Amhara, west Benishangul Gumuz, Somali, Afar, north, and northeast SNNPR. On the other hand, a low proportion of not having ANC was found in Tigray, Addis Ababa, and Dire Dawa (Figure 1).

### 3.4. Spatial Autocorrelation of Not Having ANC Visits

**Figure 2:**
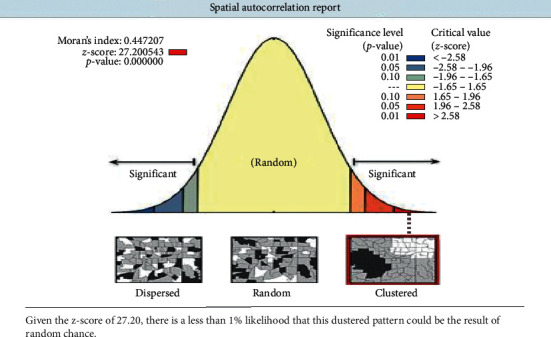
Spatial autocorrelation analysis of not having ANC visits in Ethiopia, EDHS 2016.

The clustered patterns (on the right sides) showed that high rates of not having ANC utilization were observed. The *z* value showed that there is a clustered pattern with the probability of a chance <1%. The bright red and blue colors to the end tails indicated that there is an increased significance level (Figure 2).

### 3.5. Incremental Autocorrelation of Not Having ANC Visits

**Figure 3:**
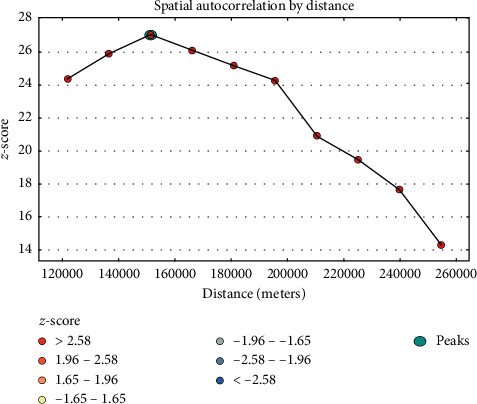
Incremental autocorrelation of not having ANC visits in Ethiopia, EDHS 2016.

Incremental spatial autocorrelation for a series of distance presented by line graph with corresponding z-score was done to determine the average nearest neighbor and minimum and maximum distance band. A total of 10 distance bands were detected by a beginning distance of 121,803 meters, and the first maximum peak (clustering) was observed at 151,378.64 meters (Figure 3).

### 3.6. Hot Spot Analysis of Not Having ANC Visits

**Figure 4:**
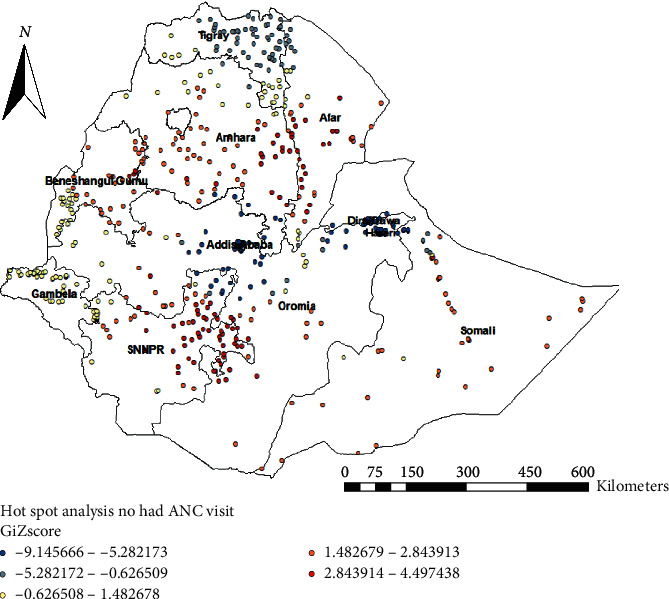
Hot spot analysis of not having ANC visits in Ethiopia, EDHS 2016.

Hot spot analysis was performed to identify high-risk areas of not having ANC visits in Ethiopia. The red color indicates significant risky areas and it is found in the central and southern parts of Amhara, eastern SNNPR, northeast Somali, Afar, and Benishangul Gumuz regions, whereas the blue color indicates less risky areas of not having ANC visits and is observed in Tigray, Addis Ababa, Harari, and Dire Dawa (Figure 4).

### 3.7. Interpolation of Not Having ANC Visits

**Figure 5:**
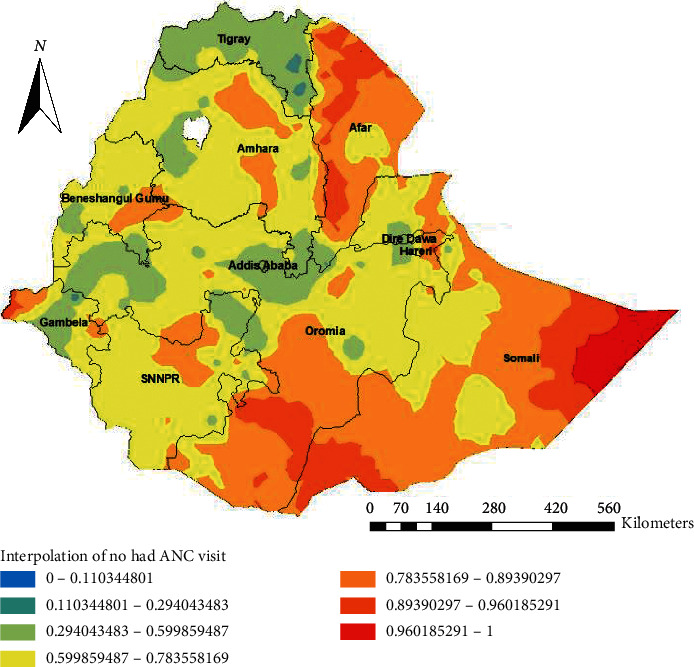
Interpolation of not having ANC visits in Ethiopia, EDHS 2016.

As we move from the blue- to the red-colored areas, the possibility of not having an ANC visit was increasing. The red color predicts high-risk areas of not having ANC and the blue and semi-blue colors predict low-risk areas of not having ANC in Ethiopia. Tigray, Oromia, and southern Amhara were predicted as more risky areas compared to other regions. In contrast, the green color indicates fewer risk areas for not having ANC visits and it was observed in Tigray, Addis Ababa, Oromia, and Dire Dawa (Figure 5).

### 3.8. Spatial SaTScan Analysis of Not Having ANC Utilization

**Figure 6:**
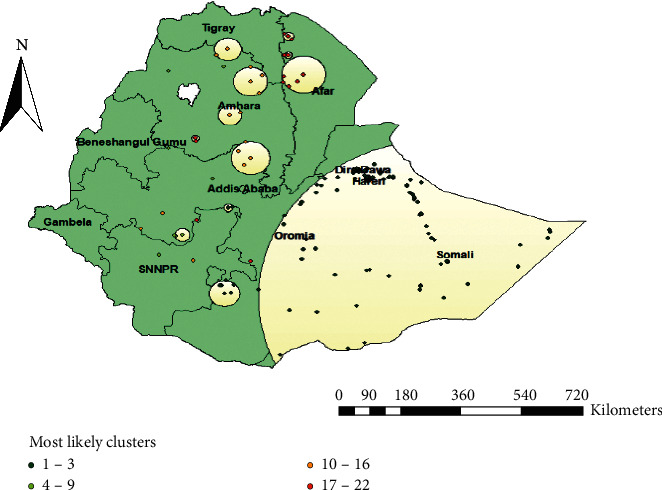
Spatial scan analysis of not having ANC visits in Ethiopia, EDHS 2016.

A total of 161 significant clusters were identified, of which 152 were most likely (primary) clusters and 6 were secondary clusters. The primary clusters' spatial window was located in Somalia, Oromia, Afar, Dire Dawa, and Harari with LLR of 133.02, at *p* < 0.001. This indicates women within the spatial window had 1.76 times higher risk of not having ANC visits compared with women outside the window. The secondary clusters' spatial window was typically located in Western Afar that was centered at ((5.725346 N, 38.264767 E)/44.75 km) with a 44.75 km radius and LLR of 49 at *p*-value <0.001. This means women within the spatial window had 1.87 times higher risk of not having ANC visits than outside the window (Table 3 and Figure 6).

### 3.9. Model Diagnosis

The model with the highest log-likelihood ratio test (−3105.43) and with the smallest deviance was used as the best-fitted model (Table 4).

### 3.10. Multilevel Logistic Regression

Bivariable multilevel logistic regression analysis was conducted to identify variables that were significant at a *p*-value of <0.2. In the multilevel logistic regression analysis, individual-level factors such as religion, maternal education, husband education, wealth index, birth order, parity, and distance from the health institution were found to be significantly associated with not having ANC visits. Also, community-level factors like place of residence, region, community distance from the health institution, and community media exposure were statistically significant factors of not having ANC.

The odds of not having ANC visits among Muslim women were reduced by 30% as compared to Orthodox Christians (AOR = 0.70 : 95% CI 0.52, 0.96). The odds of not having ANC among women of other religions (traditional and Catholic) were 2.5 times higher than Orthodox religion followers (AOR = 2.5 : 95% CI 1.51, 4.20).

Primary, secondary, and higher education of women have reduced the odds of not having ANC by 41% (AOR = 0.59 : 95% CI 0.49, 0.71), 55% (AOR = 45 : 95% CI 0.30, 0.70), and 75% (AOR = 15 : 95% CI 0.06, 0.38), respectively, as compared to women who were unable to read and write. Husbands' education level being primary and secondary has reduced the odds of not having ANC by 32% and 50%, respectively, compared to men who are unable to read and write (AOR = 0.68 : 95% CI 0.57, 0.80) (AOR = 0.50 : 95% CI 0.37, 0.67).

The odds of not having ANC visits among women who answered that distance from any health institution is a big problem were reduced by 24% (AOR = 0.76 95% CI 0.65,0.89) as compared to women who answered that distance from health institution was not a big problem. The odds of not having ANC were increased by 35% (AOR = 1.35: (95%CI, 1.03, 1.76)) and 56% (AOR = 1.56: (95%CI, 1.09,2.23)) among women who had 2 to 4 and more than five birth orders compared to one birth order, respectively. The odds of not having ANC visits among women of middle and rich wealth status were decreased by 29% (AOR = 0.71: (95%CI, 0.58, 0.88)) and 35% (AOR = 0.65: (95%CI, 0.51, 0.82)), respectively, compared to poor women.

Women in rural clusters were 2.38 (AOR = 2.38 : 95%, 1.54, 3.66) times more likely to not have ANC visits than women in urban clusters. Regarding regions, women living in Afar (AOR = 4.10, (95% CI, 1.54,10.93)), Oromia (AOR = 3.95, (95% CI, 1.44, 9.63)), Somali (AOR = 5.20, (95% CI 2.55, 13.61)), and Gambella (AOR = 2.95, (95% CI 1.11,7.85)) had higher odds of not having ANC visits five years preceding the survey as compared to Addis Ababa. The odds of not having ANC visits among women living in Tigray (AOR = 0.35: (95% CI, 0.13, 0.96) were reduced by 65% as compared to Addis Ababa.

Women in the cluster who answered that distance from the health institution was not a big problem had a decrease in ANC visits by 24% (AOR = 0.76, (95%CI, 0.58, 0.99)) than women who answered distance from health institution was a big problem. Furthermore, the odds of having no ANC among women with high media exposure in the cluster (community) were reduced by 42% (AOR = 0.68, (95% CI, 0.52, 0.89)) compared to women with low media exposure (Table 5).

## 4. Discussion

This study tried to address both individual- and community-level determinants of not having ANC in Ethiopia based on EDHS 2016. Individual factors that affect not having ANC were religion, mother's education, husband's education, and distance as a big problem, birth order, and wealth index. Community factors such as region, community distance as a big problem, community media exposure, and community women education significantly affected women not having ANC.

Being a follower of Islam religion decreased the odds of not having ANC compared with Orthodox Christians. This study is in line with a study conducted in Nigeria [33]. Moreover, a study from Southeastern Nigeria showed that religion did not affect ANC utilization [34]. This difference could be due to the large sample used in the recent study and a small sample used in the previous study.

Female education increases the odds of not having ANC as compared with women who are unable to read and write. This study is in line with a study conducted in Bangladesh [10] and Pakistan [35]. The difference could be explained by the fact that women who are unable to read and write were more likely associated with inequalities in service delivery care [36]. The other reason could be that educated women had much higher self-reliance on choosing reproductive health and have great potential to decide freely on where and when to seek medical services regardless of husband's approval [37]. Similarly, women whose husbands have acquired primary and secondary education increased the odds of not having ANC as compared with husbands who were unable to read and write. This finding is in line with a study conducted in several Sub-Saharan Africa (SSA) countries [10] and Afghanistan 2015 Demographic and Health Survey [9].

Women who felt the distance from the health center is a big problem had decreased the odds of not having ANC compared with women who felt it was a small problem. Likewise, at the community level, women who felt community distance from the health institution is a big problem had decreased the odds of not having ANC compared with women who did not feel that. This finding is supported by the Nigerian Demographic and Health Survey data 2013 [38].

Birth order is another important predictor of not having ANC in this study. Accordingly, women whose birth order 2–4 and ≥5 years increased the odds of not having ANC compared with birth order of 1 year. This study is in line with a study conducted in EDHS 2011 [39]. The finding is also supported by the Indonesia 2007 Demographic and Health Survey [40].

Having a middle and rich wealth index decreased the odds of not having ANC as compared with poor wealth index women. This finding is in line with a multiple indicator cluster survey conducted in Pakistan [35] and Indonesia [40]. This could be because poor women had financial barriers to the utilization of ANC and the difficulty of addressing wealth-related inequality [41]. Besides, trend analysis in the Benishangul Gumuz region from 2000 to 2011 showed that ANC coverage among poor women was lower than rich women [13]. This inequality among different economic strata provides basic information to highlight the need to put more resources to poor households. Another study from Benin also showed that economically disadvantaged women had lower ANC coverage than women in rich wealth index [42].

Residing in rural residence increased the odds of not having ANC compared with women of urban counterparts. This finding is in agreement with a study from Nigeria [38], 2015–2016 Indian Demographic and Health Survey [43], and Benishangul region [11]. This difference could be explained by inequalities in service accessibility and delivery in the rural setup compared with urban counterparts [44].

In Ethiopia, the distribution of ANC is non-random. Oromia, Somalia, and Gambella regions had higher odds of not having ANC. This finding is in agreement with a previous study conducted in EDHS 2000–2011. According to the study, it was shown that the distribution of ANC among pregnant women in Ethiopia is quite different across the country. Besides, this finding is in agreement with a study conducted in India, in which ANC is affected by regional variation [45]. This study is also supported by a study from Indonesia [46]. This could be due to an imbalance of demand and supply like poor infrastructure and poor quality in service delivery. Therefore, characterizing the spatial distribution of ANC has a paramount significance to identify hot spot areas and to provide spatially targeted interventions. This could further help to tackle inequalities in service delivery among different regions and to provide a fair distribution of quality health care.

Women with high community media exposure decreased the odds of not having ANC compared with women having community media exposure. This study is in line with a study from Ethiopia based on EDHS 2011 [47]. It could be that the mass media has the potential to reach many people at a time and increase knowledge by changing family behavior on health outcomes [48]. This finding is also supported by a study conducted in Malawi [49] and Nigeria [38]. This could further improve the overall maternal health outcome in pregnant women.

The strength of this study was its representativeness at the regional and national levels in Ethiopia and it included spatial analysis to show the spatial pattern of not having ANC visits. Hence, we can provide a generalization to the entire nation. However, the study has significant limitations. Since we use a cross-sectional study, we could not establish a cause-effect relationship. Recall bias could be the second limitation in this study because data was collected five years preceding the survey. Thirdly, the quality of ANC data was not collected; as a result, we could not ascertain whether women missed the service due to poor quality or not.

The study has significant importance for policymakers and planners. It might provide valuable information on regional differences and this will create a social justice issue. Hence, stakeholders will take prompt interventions for disadvantageous regions. Therefore, inequality in ANC service delivery may be addressed. Finally, this study will be important to the Ministry of Health, different regional health bureaus, nongovernmental organizations, and any interested stakeholders to design and commence appropriate intervention strategies to improve ANC utilization in Ethiopia.

## 5. Conclusion

The spatial distribution of ANC in Ethiopia is non-random. A higher proportion of not having ANC is found in northeast Amhara, west Benishangul Gumuz, Somali, Afar, north, and northeast SNNPR. On the other hand, a low proportion of not having ANC was found in Tigray, Addis Ababa, and Dire Dawa. In Ethiopia, being a follower of the Islam religion, mother's primary and secondary level education, husband's education, distance from the health facility being a big problem, middle and rich wealth index, residents of Tigray, and high community media exposure have reduced the odds of not having ANC. In contrast, high birth order and regions of Oromia, Gambella, and Somalia increased the odds of not having ANC in Ethiopia.

## Figures and Tables

**Table 1:** Socioeconomic characteristics of women, 15–49 years, in Ethiopia, 2016 EDHS.

Variables	Frequency (%)	Percentages
ANC visit		
No	2,792	37.42
Yes	4,670	62.58
Mother's age		
<20 years	691	9.26
20–34 years	4845	64.93
35–49 years	1926	25.81
Mean ± SD	29+-9.15	
Sex of the household head		
Male	6360	85.23
Female	1102	14.77
Marital status		
Not having a partner	381	5.10
Having a partner	7081	94.90
Religion		
Orthodox	2826	37.87
Muslim	2789	37.37
Protestant	1621	21.72
Others^*∗*^	226	3.03
Residence		
Urban	961	12.88
Rural	6501	87.12
Region		
Tigray	696	6.96
Afar	71	0.95
Amhara	1601	21.45
Oromia	3098	41.52
Somalia	267	3.85
Benishangul Gumuz	80	1.07
SNNP	1558	20.88
Gambella	20	0.27
Harari	17	0.23
Addis Ababa	197	2.64
Dire Dawa	33	0.44
Educational status of the mother		
Unable to read and write	4705	63.05
Primary education	2113	28.31
Secondary education	415	5.56
Higher education	229	3.08
Educational status of the husband		
Unable to read and write	3327	47.63
Primary education	2679	38.36
Secondary education	606	8.67
Higher education	373	5.34
Occupational status of the mother		
Not working	4014	53.79
Working	3448	46.21
Occupational status of the husband		
Not working	4129	55.34
Working	3333	44.66
Distance to health institution		
A big problem	4336	58.11
Not a big problem	3126	41.89
Media exposure		
No media exposure	4914	67.02
Has media exposure	2418	32.98
Birth order		
1	1408	18.87
2–4	3137	42.04
≥ 5	2917	39.09
Parity		
≤ 2	2576	34.43
2–5	2795	37.45
≥ 5^+^	2091	28.02
Wealth index		
Poor	3248	43.53
Middle	1553	20.08
Rich	2661	36.66

Others^*∗*^: Catholic and traditional followers.

**Table 2:** Community-level characteristics of women, 15–49 years, in Ethiopia from January 18 to June 27, 2016.

Community variables	Frequency	Percent (%)
Residence		
Rural	441	68.68
Urban	202	31.32
Region		
Tigray	63	9.80
Afar	53	8.24
Amhara	71	11.04
Oromia	74	11.51
Somali	67	10.42
Benishangul	50	7.78
SNNPR	71	11.04
Gambella	50	7.78
Harari	44	6.84
Addis Ababa	56	8.71
Dire Dawa	44	6.84
Community distance to a health facility		
A big problem	314	48.83
Not a big problem	329	51.17
Community media exposure		
Low	321	49.92
High	322	50.08
Community poverty status		
High	319	49.61
Low	324	50.39
Community women educational attainment		
Low	318	49.46
High	325	50.54

**Table 3:** SaTScan analysis of ANC utilization of women who gave birth in the last five years in Ethiopia, EDHS 2016.

Cluster	EA (enumeration area)	Coordinate or radius	Rr	LLR	*p*-value
Primary (152)	138, 164, 85, 358, 146, 492, 92, 490, 543, 278, 171, 198, 95, 318, 77, 187, 497, 556, 520, 629, 521, 588, 553, 458, 480, 208, 214, 251, 573, 239, 269, 116, 22, 394, 378, 630, 568, 33, 277, 286, 527, 289,64, 439, 57, 186, 8, 210, 472, 452, 377, 454, 513, 436, 501, 212, 68, 580, 622, 483, 566, 133, 587, 194, 240, 500, 321, 418, 58, 115, 29,44, 534, 179, 257, 387, 157, 397, 56, 607, 228, 28, 614, 396, 60,393, 357, 419, 443, 173, 238, 329, 1, 288, 383, 495, 381, 610, 473, 372, 453, 242, 523, 281, 642, 166, 311, 307, 30, 557, 202, 441, 594, 613, 352, 74, 519, 380, 535, 273, 471, 631, 151, 5, 185, 444, 111, 514, 282, 27, 390, 606, 493, 385, 224, 467, 644, 43, 363, 190, 546,101, 140, 25, 93, 7, 476, 412, 529, 245, 123, 333, 506, 319, 422	(5.589269 N, 44.175034 E)/552.72 km	1.76	133.02	<0.001

Secondary (6)	21, 398, 182, 574, 316, 232	(5.725346 N, 38.264767 E)/44.75 m	1.87	49.10	<0.001

**Table 4:** Model comparison and goodness of fit test in the multilevel analysis.

Model	Model I	Model II	Model III	Model IV
Log-likelihood (LL)	−3815.22	−3224.13	−3546.81	−3105.43
Deviance	7630.44	6448.26	7093.62	6210.86

**Table 5:** Multivariable multilevel logistic regression analysis result of both individual- and community-level factors associated with ANC visits in Ethiopia, EDHS 2016.

Individual- and community-level factors	Models			
	Null model AOR (95%CI)	Model II AOR (95%CI)	Model III AOR (95%CI)	Model IV AOR (95%CI)
Mother's age				
<20 years		1		1
20–34 years		0.72(0.55,0.94)		0.81 (0.62,1.06)
35–49 years		0.85(0.61,1.19)		1.07 (0.77, 1.50)
Household head				
Male		1		1
Female		1.17(0.97,1.41)		1.13 (0.94, 1.37)
Religion				
Orthodox		1		1
Muslim		1.62 (1.26,2.07)		0.70 (0.52, 0.96)^*∗*^
Protestant		2.15 (1.63,2.84)		1.36 (0.99, 1.87)
Others^*∗*^		4.01 (2.41,6.66		2.52 (1.51,4.20)^*∗∗*^
Mother's education				
Unable to read and write		1		1
Primary education		0.53 (0.44,0.64)		0.59 (0.49, 0.71)^*∗∗∗*^
Secondary education		0.34 (0.22,0.51)		0.45 (0.30, 0.70)^*∗∗∗*^
Higher education		0.095 (0.038,0.23))		0.15 (0.06, 0.38)^*∗∗∗*^
Husband's education				
Unable to read and write		1		1
Primary education		0.63 (0.53,0.74)		0.68 (0.57,0.80)^*∗∗∗*^
Secondary education		0.44 (0.33,0.60)		0.50 (0.37, 0.67)^*∗∗∗*^
Higher education		0.63 (0.43,0.94)		0.70 (0.47, 1.05)
Mother's occupation				
Working		1		1
Not working		1.13 (0.96,1.31)		1.07 (0.92, 1.26)
Husband's occupation				
Working		1		1
Not working		1.02 (0.85,1.31)		0.99 (0.82, 1.19)
Distance to HF				
Not a big problem		1		1
A big problem		0.65 (0.56,0.76)		0.76 (0.65, 0.89)^*∗∗*^
Media exposure				
No media exposure		1		1
Has media exposure		0.83 (0.70, 0.99)		0.93 (0.78, 1.11)
Birth order				
1		1		1
2–4		1.36 (1.04, 1.78)		1.35 (1.03, 1.76)^*∗∗∗*^
≥ 5		1.59 (1.12, 2.27)		1.56 (1.09, 2.23)^*∗∗*^
Parity				
≤ 2		1		1
2–5		1.02 (0.82, 1.28)		0.98 (0.78, 1.23)
≥ 5^+^		1.13(0.81, 1.56)		1.01 (0.73, 1.40)
Wealth index				
Poor		1		1
Middle		0.65 (0.53, 0.79)		0.71 (0.58,0.88)^*∗∗*^
Rich		0.46 (0.37, 0.58)		0.65(0.51, 0.82)^*∗∗*^
Residence				
Urban			1	1
Rural			3.44 (2.28, 5.19)	2.38(1.54, 3.66)^*∗∗∗*^
Region				
Addis Ababa			1	1
Tigray			0.42 (0.184, 0.97)	0.35 (0.13,0.96)^*∗*^
Afar			4.16 (2.04, 10.39)	4.10 (1.54., 10.93)^*∗*^
Amhara			1.96 (0.88, 4.39)	1.58 (0.60, 4.14)
Oromia			3.95 (1.79, 8.71)	3.73 (1.44,9.63)^*∗*^
Somali			6.00 (2.72, 13.22)	5.20 (2.55,13.61)^*∗*^
Benishangul			1.43 (0.63,3.27)	1.38 (.52, 3.66)
SNNPR			1.57 (0.71, 3.48)	1.22 (0.47, 3.20)
Gambella			3.83 (1.71, 8.60)	2.95 (1.11,7.85)^*∗*^
Harari			2.12 (0.91, 4.94)	2.64(0.96, 7.23)
Dire Dawa			0.95(0.39,2.28)	1.07(0.37,3.02)
Community distance to HF				
A big problem			1	1
Not a big problem			0.67 (0.52, 0.87)	0.76 (0.58,0.99)^*∗*^
Community media exposure				
Low			1	1
High			0.58 (0.44, 0.76)	0.68 (0.52, 0.89)^*∗*^
Community poverty status				
High			1	1
Low			0.70(0.52, .93)	0.92 (0.68,1.24)
Community women education				
Low			1	1
High			0.52 (0.40, 0.68)	0.64 (0.49, 0.83)^*∗*^
Random effects				
ICC	0.48.(0.43, 0.52)			
PCV	Ref	59%	69%	72%
MOR	5.30	2.90	2.51	2.38

Others^*∗*^: Catholic and traditional religion, AOR : adjusted odds ratio, and HF : health facility. ^*∗*^*p* value ≤ 0.05, ^*∗∗*^*p* value = 0.01, ^*∗∗∗*^*p* value = 0.001.

## Data Availability

The data used in this study are publicly available at http://www.measuredhs.com, aggregated secondary data, which have no personal identifying information that can be linked to the study participants. The confidentiality of the data was maintained anonymously.

## Abbreviations

AIC: Akaike Information Criteria
ANC: Anti-Natal Care
AOR: Adjusted odds ratio
BIC: Bayesian Information Criteria
DHS: Demographic and Health Survey
EA: Enumeration area
EDHS: Ethiopian Demographic and Health Survey
ICC: Intraclass Correlation Coefficient
LBW: Low birth weight
LL: Likelihood
MOR: Median odds ratio
PCV: Proportional change in variance
SD: Standard deviation
SNNP: Southern Nation and Nationality of People
SSA: Sub-Saharan Africa
VIF: Variance Inflation Factor
WHO: World Health Organization.

## References

[1] Tikmani S. S., Ali S. A., Saleem S. (2019). Trends of antenatal care during pregnancy in low- and middle-income countries: findings from the global network maternal and newborn health registry. *Seminars in Perinatology*.

[2] Ataguba J. E. (2018). A reassessment of global antenatal care coverage for improving maternal health using sub-Saharan Africa as a case study. *PloS One*.

[3] Getahun H., Matteelli A., Abubakar I. (2015). Management of latent *Mycobacterium tuberculosis* infection: WHO guidelines for low tuberculosis burden countries. *The European Respiratory Journal*.

[4] Gonçalves L. C., Endrass J., Rossegger A., Dirkzwager A. J. (2016). A longitudinal study of mental health symptoms in young prisoners: exploring the influence of personal factors and the correctional climate. *BMC Psychiatry*.

[5] Forrester A., Hopkin G. (2019). Mental health in the criminal justice system: a pathways approach to service and research design. *Criminal Behaviour and Mental Health*.

[6] Martin M. S., Crocker A. G., Potter B. K., Wells G. A., Grace R. M., Colman I. (2018). Mental health screening and differences in access to care among prisoners. *The Canadian Journal of Psychiatry*.

[7] Agency C. S. (2016). Central statistical agency (CSA) [Ethiopia] and ICF. *Ethiopia Demographic and Health Survey 2016*.

[8] Ayalew T. W., Nigatu A. M. (2018). Focused antenatal care utilization and associated factors in Debre Tabor Town, northwest Ethiopia, 2017. *BMC Research Notes*.

[9] Azimi M. W., Yamamoto E., Saw Y. M (2019). Factors associated with antenatal care visits in Afghanistan: secondary analysis of Afghanistan Demographic and Health Survey 2015. *Nagoya Journal of Medical Science*.

[10] Woldegiorgis M. A., Hiller J., Mekonnen W., Meyer D., Bhowmik J. (2019). *Determinants of Antenatal Care and Skilled Birth Attendance in Sub-saharan Africa: A Multilevel Analysis*.

[11] Tiruaynet K., Muchie K. F. (2019). Determinants of utilization of antenatal care services in benishangul gumuz region, Western Ethiopia: a study based on demographic and health survey. *BMC Pregnancy and Childbirth*.

[12] Fekede B. (2007). Antenatal care services utilization and factors associated in Jimma Town (south west Ethiopia). *Ethiopian Medical Journal*.

[13] G/Mariam E. A., Calderon-Margalit R. (2013). Disparities in the use of antenatal care service in Ethiopia over a period of fifteen years. *BMC Pregnancy and Childbirth*.

[14] Acharya D., Singh J. K., Kadel R., Yoo S. J., Park J. H., Lee K. (2018). Maternal factors and utilization of the antenatal care services during pregnancy associated with low birth weight in rural Nepal: analyses of the antenatal care and birth weight records of the MATRI-SUMAN trial. *International Journal of Environmental Research and Public Health*.

[15] Teklay G., Teshale T., Tasew H., Mariye T., Berihu H., Zeru T. (2018). Risk factors of preterm birth among mothers who gave birth in public hospitals of central zone, Tigray, Ethiopia: unmatched case-control study 2017/2018. *BMC Research Notes*.

[16] John A. E., Nilima, Binu V. S., Unnikrishnan B. (2019). Determinants of antenatal care utilization in India: a spatial evaluation of evidence for public health reforms. *Public Health*.

[17] Kim K. H., Choi J. W., Oh J., Moon J., You S., Woo Y. (2019). What are the barriers to antenatal care utilization in rufisque district, Senegal?: a bottleneck analysis. *Journal of Korean Medical Science*.

[18] Exavery A., Kante A. M., Hingora A., Mbaruku G., Pemba S., Phillips J. F. (2013). How mistimed and unwanted pregnancies affect timing of antenatal care initiation in three districts in Tanzania. *BMC Pregnancy and Childbirth*.

[19] Tura H., Licoze A. (2019). Women’s experience of intimate partner violence and uptake of Antenatal Care in Sofala, Mozambique. *PloS One*.

[20] Yaya S., Uthman O. A., Amouzou A., Ekholuenetale M., Bishwajit G. (2018). Inequalities in maternal health care utilization in Benin: a population based cross-sectional study. *BMC Pregnancy and Childbirth*.

[21] Mbuagbaw L., Medley N., Darzi A. J., Richardson M., Habiba Garga K., Ongolo-Zogo P. (2015). Health system and community level interventions for improving antenatal care coverage and health outcomes. *The Cochrane Database of Systematic Reviews*.

[22] Okedo-Alex I. N., Akamike I. C., Ezeanosike O. B., Uneke C. J. (2019). Determinants of antenatal care utilisation in sub-Saharan Africa: a systematic review. *BMJ Open*.

[23] El-Gilany A., Khater M., Gomaa Z. (2016). Psychiatric Disorders among Prisoners: a National Study in Egypt HIV and tuberculosis in prisons in sub-Saharan Africa. *East Asian Archives Of Psychiatry*.

[24] Waldhor T. (1996). *The Spatial Autocorrelation Coefficient Moran’s I under Heteroscedasticity*.

[25] Kulldoff M. (2018). *SaTScan User Guide*.

[26] Goldstein H. (2011). *Multilevel Statistical Models*.

[27] Hox J. J., Moerbeek M., Van de Schoot R. (2017). *Multilevel Analysis: Techniques and Applications*.

[28] Rabe-Hesketh S., Skrondal A. (2008). *Multilevel and Longitudinal Modeling Using Stata*.

[29] Tom A., Bosker T. A. S. R. J., Bosker R. J. (1999). *Multilevel Analysis: An Introduction to Basic and Advanced Multilevel Modeling*.

[30] Heinrich C. J., Lynn L. E. (2001). Means and ends: a comparative study of empirical methods for investigating governance and performance. *Journal of Public Administration Research and Theory*.

[31] Merlo J., Chaix B., Yang M., Lynch J., Råstam L. (2005). A brief conceptual tutorial of multilevel analysis in social epidemiology: linking the statistical concept of clustering to the idea of contextual phenomenon. *Journal of Epidemiology & Community Health*.

[32] Merlo J., Chaix B., Yang M., Lynch J., Råstam L. (2005). A brief conceptual tutorial on multilevel analysis in social epidemiology: interpreting neighbourhood differences and the effect of neighbourhood characteristics on individual health. *Journal of Epidemiology & Community Health*.

[33] Al-Mujtaba M., Cornelius L. J., Galadanci H. (2016). Evaluating religious influences on the utilization of maternal health services among muslim and christian women in north-Central Nigeria. *BioMed Research International*.

[34] Nwosu B. O., Ugboaja J. O., Obi-Nwosu A. L., Nnebue C. C., Ifeadike C. O. (2012). Proximate determinants of antenatal care utilization among women in southeastern Nigeria. *Nigerian Journal of Medicine: Journal of the National Association of Resident Doctors of Nigeria*.

[35] Ghaffar A., Pongponich S., Ghaffar N., Mehmood T. (2015). Factors associated with utilization of antenatal care services in Balochistan province of Pakistan: an analysis of the multiple indicator cluster survey (MICS) 2010. *Pakistan Journal of Medical Sciences*.

[36] Myint A. N. M., Liabsuetrakul T., Htay T. T., Wai M. M., Sundby J., Bjertness E. (2018). Inequity in the utilization of antenatal and delivery care in Yangon region, Myanmar: a cross-sectional study. *International Journal for Equity in Health*.

[37] Umar A. S. (2017). Does female education explain the disparity in the use of antenatal and natal services in Nigeria? Evidence from demographic and health survey data. *African Health Sciences*.

[38] Adewuyi E. O., Auta A., Khanal V. (2018). Prevalence and factors associated with underutilization of antenatal care services in Nigeria: a comparative study of rural and urban residences based on the 2013 Nigeria demographic and health survey. *PloS One*.

[39] Yaya S., Bishwajit G., Ekholuenetale M., Shah V., Kadio B., Udenigwe O. (2017). Timing and adequate attendance of antenatal care visits among women in Ethiopia. *PloS One*.

[40] Kamal S. M. M., Hassan C. H., Islam M. N. (2015). Socioeconomic factors associated with contraceptive use and method choice in urban slums of bangladesh. *Asia Pacific Journal of Public Health*.

[41] Jalloh M. B., Bah A. J., James P. B., Sevalie S., Hann K., Shmueli A. (2019). Impact of the free healthcare initiative on wealth-related inequity in the utilization of maternal & child health services in Sierra Leone. *BMC Health Services Research*.

[42] Dansou J., Adekunle A., Arowojolu A. (2017). Factors associated with antenatal care services utilisation patterns amongst reproductive age women in Benin Republic: an analysis of 2011/2012 Benin republic’s demographic and health survey data. *Nigerian Postgraduate Medical Journal*.

[43] Singh L., Dubey R., Singh S., Richagoel, Nair S., Singh P. K. (2019). Measuring Quality of Antenatal Care: a secondary analysis of national survey data from India. *BJOG: An International Journal of Obstetrics and Gynaecology*.

[44] Telisinghe L., Charalambous S., Topp S. M. (2016). HIV and tuberculosis in prisons in sub-Saharan Africa. *The Lancet*.

[45] Ponna S. N., Upadrasta V. P., Babu Geddam J. J., Dudala S. R., Sadasivuni R., Bathina H. (2017). Regional variation in utilization of antenatal care services in the state of Andhra Pradesh. *Journal of Family Medicine and Primary Care*.

[46] Tripathi V., Singh R. (2017). Regional differences in usage of antenatal care and safe delivery services in Indonesia: findings from a nationally representative survey. *BMJ Open*.

[47] Assefa E., Tadesse M. (2017). Factors related to the use of antenatal care services in Ethiopia: application of the zero-inflated negative binomial model. *Women & Health*.

[48] Sarrassat S., Meda N., Badolo H. (2018). Effect of a mass radio campaign on family behaviours and child survival in Burkina Faso: a repeated cross-sectional, cluster-randomised trial. *The Lancet Global Health*.

[49] Zamawe C. O. F., Banda M., Dube A. N. (2016). The impact of a community driven mass media campaign on the utilisation of maternal health care services in rural Malawi. *BMC Pregnancy and Childbirth*.

